# Complement Activation in Liver Transplantation: Role of Donor Macrosteatosis and Implications in Delayed Graft Function

**DOI:** 10.3390/ijms19061750

**Published:** 2018-06-13

**Authors:** Kelley Núñez, Paul Thevenot, Abeer Alfadhli, Ari Cohen

**Affiliations:** Institute of Translational Research, Ochsner Health System, New Orleans, LA 70121, USA; kelley.nunez@ochsner.org (K.N.); paul.thevenot@ochsner.org (P.T.); v-aafadhli@ochsner.org (A.A.)

**Keywords:** complement, extended criteria donor, liver transplantation, steatosis

## Abstract

The complement system anchors the innate inflammatory response by triggering both cell-mediated and antibody-mediated immune responses against pathogens. The complement system also plays a critical role in sterile tissue injury by responding to damage-associated molecular patterns. The degree and duration of complement activation may be a critical variable controlling the balance between regenerative and destructive inflammation following sterile injury. Recent studies in kidney transplantation suggest that aberrant complement activation may play a significant role in delayed graft function following transplantation, confirming results obtained from rodent models of renal ischemia/reperfusion (I/R) injury. Deactivating the complement cascade through targeting anaphylatoxins (C3a/C5a) might be an effective clinical strategy to dampen reperfusion injury and reduce delayed graft function in liver transplantation. Targeting the complement cascade may be critical in donor livers with mild to moderate steatosis, where elevated lipid burden amplifies stress responses and increases hepatocyte turnover. Steatosis-driven complement activation in the donor liver may also have implications in rejection and thrombolytic complications following transplantation. This review focuses on the roles of complement activation in liver I/R injury, strategies to target complement activation in liver I/R, and potential opportunities to translate these strategies to transplanting donor livers with mild to moderate steatosis.

## 1. Introduction

The United Network for Organ Sharing reported 7841 liver transplants performed for end-stage liver disease in 2016 [1]. Although donation and utilization increased in 2016, the year ended with 11,140 active waitlist candidates, highlighting the stable deficit between waitlist candidates and the available donor pool. Given the disparity between recipient need and graft availability, transplant centers have increasingly utilized extended criteria donor (ECD) livers to address the deficit [2,3]. The ECD designation is assigned to livers that are considered marginal for transplantation due to known risk factors affecting graft function, transplant outcomes, and risk of transmitting infection. Common ECD designations include donation with advanced age, after cardiac death, positive for hepatitis B or C, prolonged ischemia time, or having lipid accumulation (steatosis) in hepatocytes. Managing risk is a critical factor in ECD transplantation, as these organs have increased susceptibility to hypoxia and reperfusion injury with increased risk for graft loss or delayed function [4,5].

Despite ongoing efforts to manage or reduce the risks associated with ECD grafts, utilization rates have remained stable at ~25% since 2005 [6]. A recent retrospective study of patients with >15 model of end-stage liver disease (MELD) scores found that >50% of waitlist mortality patients declined at least one transplant offer [7]. The reason for offer rejection in nearly 75% of these cases was due to concerns over donor quality or age. Surprisingly, many of these livers were successfully transplanted into lower priority patients. Increasing the safe utilization of ECD livers will require clarifying the molecular pathways associated with delayed graft function (DGF) or primary non-function (PNF) as well as strategies to quantify and manage these factors without influencing patient outcomes. Identifying specific mediators will allow centers to quantitatively ascertain high versus low risk ECD livers and therapeutically neutralize mediators to control DGF/PNF risk.

Numerous reports have shown that when risks associated with ECD livers are identified and managed, these organs can be utilized with excellent patient outcomes [8,9,10]. Unfortunately, alarming trends in obesity and non-alcoholic fatty liver disease (NAFLD) [11] have already started to impact liver transplantation [1] and the donor liver pool [12,13]. The prevalence of steatosis in liver procurements ranges from 10–30% in the literature [12,13]. Obesity is a well-known risk factor for hepatic steatosis, and clinical profiles of waitlist registrants with consistent NAFLD continue to increase [1,11]. These trends suggest the prevalence of steatosis will increase in the coming years, and require centers to confidently assess and address donor steatosis or risk a widening gap between transplant candidates and available donor livers.

In ECD steatosis, careful consideration must be given to both the degree of steatosis as well as the percentage of the liver affected. Steatosis is characterized as lipid accumulation within hepatocytes and can manifest as either microsteatosis (MiS) or macrosteatosis (MaS). Steatosis can lead to hepatic endoplasmic reticulum stress and mitochondrial dysfunction. Histologically, MiS exists as small lipid-containing vesicles within hepatocytes, and grafts with MiS are considered viable even with 100% of liver affected. MaS exists as large lipid-containing vesicles, which displace the hepatocyte nucleus [14], and grafts with MaS exceeding 30% are generally not utilized due to poor outcomes [14,15,16]. MaS is graded on a scale of mild (<30%), moderate (<60%), or severe (>60%), with several retrospective studies over the past decade showing a progressive increase in PNF risk as MaS grade increases [5,16,17,18,19,20], although other metabolic-associated risk factors may play a greater role influencing transplant outcomes [21,22,23].

This review focuses on the role of complement signaling in settings of donor steatosis, specifically the interactions between steatosis and complement activation from procurement through reperfusion. It also addresses the potential for targeting complement activation in ECD steatosis. The liver is the major site for the synthesis of complement components, with complement activation playing an integral role in both injury and regeneration following ischemia/reperfusion (I/R) injury. With clinical trials currently underway targeting complement activation in kidney and liver transplantation, this review aims to review the current research on the role of complement activation in NAFLD, liver I/R injury, and aberrant complement signaling in ECD steatosis. Finally, we address strategies to target complement activation through liver storage solutions and machine perfusion systems to improve ECD steatosis viability testing and safe utilization in liver transplantation.

## 2. I/R Injury in Liver Procurement and Transplantation

I/R injury is the major cause of hepatocellular injury in liver transplantation. The incidence of early graft dysfunction, graft failure, and innate immune-mediated rejection are closely linked to the degree of I/R injury. I/R injury extends from the initial warm ischemic period during surgery, cold ischemia during storage, through the reperfusion phase after transplantation. In the absence of other liver disease pathology, the ischemic injury leads to nutrient and oxygen deprivation, followed by the more extensive reperfusion injury phase. The injury is initiated and exacerbated by antioxidant imbalance and the excess production of reactive oxygen species (ROS), which impairs stress-adaptive cellular responses such as autophagy [5,24,25]. The greatest concern over the course of I/R injury is the loss of hepatocytes, although numerous parenchymal and nonparenchymal cells are involved in the progression of I/R injury at different time points in the hours following reperfusion. The biomolecular responses and cellular interactions elicited by liver I/R injury have been extensively reviewed in the literature. The signaling pathways and molecular mediators driving each phase of liver I/R injury will be summarized in this section, followed by a comprehensive review of the complement system in each phase.

### 2.1. Ischemic Phase—Organ Procurement and Cold Storage

Although cold storage effectively reduces metabolic activity, hypoxia rapidly triggers metabolic adaptation immediately after blood flow is interrupted during procurement. Cold preservation solutions have been formulated and revised to limit destructive metabolic shifts (reviewed in [26]). Decades of research in cold storage solutions for liver transplantation has shown that the tissue is starved of oxygen and nutrients, halting cellular metabolism and ATP production. ATP depletion then leads to accelerated glycolysis, thus increasing lactate formation and the loss of transcellular electrolyte gradients, as well as disturbed sodium and calcium ion homeostasis resulting in calcium influx. Acidic metabolites continue to accumulate, causing metabolic acidosis and Na^+^/K^+^ ATPase transmembrane pump failure, which occurs in parallel with defective mitochondrial oxidative phosphorylation and leads to ROS accumulation. The cumulative effect of the cold ischemic phase results in activation of the endoplasmic reticulum (ER) stress response, constricted microcirculation, hepatocyte swelling, and potentially lysis, leading to necrosis [27,28].

Several studies have modeled the effects on hypoxia on hepatocytes and Kupffer cells with the goal of identifying specific mediators linked to stress-response or cell turnover, which ultimately trigger reperfusion injury [29]. These studies have identified calcium overload as a direct consequence of ATP depletion as the initial consequence of hypoxia, triggering both stress-adaptive responses and the release of molecules signaling cellular injury. Cell-to-cell injury signaling occurs through the recognition of damage-associated molecular patterns (DAMPs). Hepatocyte-derived DAMPs have emerged as one of the primary links between ischemia and reperfusion injury stages. The high-mobility group box 1 (HMGB1)-Toll-like receptor 4 (TLR4) pathway is perhaps the most characterized DAMP-sensing pathway in liver I/R injury, owing to several tissue and cell-specific TLR4 knockout models [30,31,32,33,34]. The influx and accumulation of sodium and calcium following ATPase pump failure disturbs calcium homeostasis, causing hepatocytes to swell and release HMGB1 [32,33]. HMGB1 release abrogates the homeostatic influence of intracellular HMGB1 and triggers a feed-forward mechanism of TLR4 activation. HMGB1 is released shortly after perfusion and oxygen levels are restored [29,32,34]. Lineage-specific TLR4 knockouts confirmed hepatocytes as the primary source of HMGB1 production during I/R injury [32].

Kupffer cells, although having an innately higher threshold to hypoxia compared with hepatocytes, have a similar hypoxic response to hepatocytes, which is characterized by rapid intracellular calcium flux, elevated intracellular ROS, and increased TLR4 expression during hypoxia [35,36]. Calcium sensor responses through stromal interaction molecule 1 (STIM1) trigger adaptive/inflammatory signaling pathways through nuclear factor kappa-light-chain-enhancer of activated B cells (NF-κB) with increases in transcripts encoding for inflammatory mediators: tumor necrosis factor α (TNFα), interleukin-6 (IL-6), interleukin-1 beta (IL-1β), cyclooxygenase 2 (COX2), and inducible nitric oxide synthase (iNOS). Parallel activation by DAMPs signaling through TLR4 magnifies this response as TLR4 signaling converges upon the NF-κB axis. 

Although these mechanistic studies implicate HMGB1-TLR4 and calcium overload as initial hypoxia-dependent responses that will trigger reperfusion injury during reoxygenation, it is important to note these studies observe significant caspase activation due to hypoxia alone. This suggests that products of cellular apoptosis and necrosis may be equally important triggers for subsequent reperfusion injury. Cumulative data suggests that the baseline mitochondrial potential and degree of loss during hypoxia ultimately determine whether hepatic cells undergo apoptosis or necrosis [37,38]. Studies suggest the greater burden of mitochondrial dysfunction and the greater likelihood of necrosis, as would be the case in hepatic steatosis.

### 2.2. Reperfusion Phase—Transplantation of the Graft

The reperfusion phase begins after vessel anastomosis and the restoration of blood flow, triggering the transition from ischemia to reperfusion injury. As the system shifts back to aerobic pathways, increased oxygen exceeds the capacity of cellular metabolism, leading to the generation of free radicals. Data suggests three main sources of free radical production: the uncoupling of the mitochondrial electron transport chain, xanthine dehydrogenase conversion to xanthine oxidase, and nicontinamide adenine dinucleotide phosphate (NADPH) oxidase activation [39,40]. The reperfusion injury is multifactorial, involving a diverse group of signaling pathways (TLR, heme-oxygenase 1 (HO-1), NF-κB, and nuclear factor erythroid 2 (Nrf2)) and inflammatory mediators (iNOS, TNFα, IL-1β, IL-6, and HMGB1), with time-dependent resident and circulating leukocyte involvement.

The major cause of dysfunction in this phase results from stress response programs triggered during hypoxia, including leukocyte chemotaxis directed by inflammatory cytokine/chemokine ligation, sterile inflammatory programs triggered by DAMP signaling, and the destructive interaction of redox species. The reperfusion injury occurs in two distinct phases [41,42,43], which are driven by Kupffer cells (peaking at 6 h after reperfusion) [33,44] and neutrophils (peaking at 24 h after reperfusion) [45]. Redox species production from Kupffer cells and neutrophils is the primary mediator of tissue injury during this phase [42,43,46], which is supported by studies where Kupffer cells were depleted prior to ischemia/cold storage and significantly reduced tissue injury [47,48].

Shortly after reperfusion/reoxygenation, surviving hepatocytes undergo redox-driven NK-κB signaling, resulting in the production of TNFα [49]. TNFα produced during the reperfusion phase has been implicated in numerous processes, with both hepatoprotective and pro-inflammatory elements [49,50]. TNFα signaling through Kupffer cells and infiltrating leukocytes occurs in tandem with HMGB1-TLR4 and hypoxic-phase activation, leading to the local production of redox species and chemokines, triggering neutrophil infiltration [50]. Endothelin-1, an inflammation-induced activation product of Kupffer cells, contributes to microcirculatory dysfunction, sinusoidal congestion, and hepatocyte necrosis [51,52]. Infiltrating neutrophils become activated to release redox species and proteases, exacerbating tissue injury [40,43]. ROS and protease activity linked to neutrophil influx correlate with the degree of hepatocyte necrosis and ATP depletion, with damage partially reversible with neutrophil depletion [42]. The burden of initial hepatocyte loss and the duration and intensity of the reperfusion response impact the functional capacity and viability of the liver in the subsequent days following I/R injury.

## 3. Mechanisms of Steatotic Graft Dysfunction after I/R Injury

The rising incidence of NAFLD is reflected by the increasing prevalence of steatosis in liver procurements [12,13]. Hepatic steatosis has emerged as one of the primary factors considered in determining graft suitability for transplantation. Several retrospective studies across multiple transplant centers reveal a correlation between elevated donor organ MaS and poor clinical outcomes in recipients, including a greater incidence of DGF/PNF and mortality [5,15,16]. In clinical practice, these findings have limited utilization for livers with greater than 30% MaS. Animal studies have confirmed that as MaS increases, the liver becomes increasingly susceptible to I/R injury, attributable to a dramatic increase in hepatocyte necrosis; the primary cause of PNF in these grafts following orthotopic liver transplantation (OLT) [53,54,55]. In the following section, the underlying steatosis-driven mechanisms leading to increased susceptibility are reviewed.

### 3.1. Mechanisms of Increased Susceptibility to Injury

Studies performed with cadaver livers as well as animal models of experimental diet and transgene-driven simple steatosis have shown a link between hepatic lipid burden and functional deficits in the electron respiratory chain [56,57,58,59,60]. Hepatic steatosis causes metabolic adaptations, including a shift toward enhanced fatty acid oxidation, tricarboxylic acid cycle induction, and oxidative phosphorylation, leading to elevated baseline ROS production [59,61,62]. The hepatic levels of mitochondrial complex I, which are responsible for generating a proton gradient across the inner mitochondrial membrane to drive ATP production, decrease as steatosis progresses from mild to moderate/severe. The loss of complex I is mirrored by a lower respiratory control ratio during cold storage, which is indicative of an increased inner mitochondrial membrane permeability [63]. This decrease leads to reduced mitochondrial capacity and limited ATP production, as well as the increased production of redox mediators through the β oxidation of free fatty acids during reperfusion. A recent study showed that promoting triglyceride catabolism to eliminate steatosis does not immediately reverse metabolic adaptation, but rather required an exogenous source of l-carnitine to reduce ROS production, increase ATP content, and decrease sensitivity to hypoxia/reoxygenation in previously steatotic hepatocytes [64].

Steatosis also affects stress-response pathways in hepatocytes, decreasing their threshold to hypoxia, as observed in the progression of NAFLD (reviewed in [65,66,67,68]). Molecular chaperones, which maintain ER homeostasis, are downregulated in steatosis and lead to the more rapid induction of ER stress and activation of downstream mechanisms linked to inflammation and cell turnover [69,70,71]. Metabolic adaptation in steatosis also shifts the balance of nuclear hormone receptors peroxisome proliferator-activated receptor (PPAR) α/γ, decreasing PPARα expression with the loss of cognate pro-survival signaling [72]. Elevated baseline and post-reperfusion complement activation has also been implicated in steatotic liver dysfunction [73].

The adaptive response triggered by increased lipid burden depletes energy stores while impairing stress responses, ultimately leaving the hepatocyte extremely vulnerable to a secondary insult. Increased hepatocyte turnover in steatotic livers subjected to I/R injury not only affects short-term liver function, but also amplifies reperfusion injury. Increased DAMP signaling through HMGB1-TLR4-NF-κB magnifies neutrophil infiltration and hepatocyte loss during reperfusion [53,74,75], which can be partially reduced through TLR4 knockout [75]. The net effect of I/R injury in the setting of advanced steatosis is an increased magnitude of hepatocyte loss with a correspondingly more dramatic reperfusion injury, yielding the graft non-viable.

### 3.2. Postreperfusion Syndrome with Steatotic Grafts

During reperfusion, post-reperfusion syndrome (PRS) can develop with an incidence rate reported from 12% to 33% [76]. PRS occurs within the first five minutes after graft reperfusion, and is marked by a decrease in arterial pressure of more than 30% from baseline. The direct role that I/R injury may have on the development of PRS remains unknown. The severity of I/R injuries and the development of PRS have often not correlated fueling debate (reviewed in [77,78]). A study investigating PRS in living donor liver transplants found that grafts with more MaS had an increased risk of developing PRS [79]. While MaS is known to increase the damage accumulated during I/R injuries, studies investigating steatosis and PRS are limited, and the direct impact of graft steatosis on PRS remains elusive.

## 4. The Complement System and Liver I/R Injury

### 4.1. Overview of the Complement System

The complement system is a component of the innate immune response to pathogens with central roles in sterile tissue injury and repair. Complement effector molecules circulate in a precursor state, and upon recognition of pathogen-associated molecular patterns (PAMPs) and/or DAMPs, become activated in a proteolytic and cascade-like fashion. The complement system can be activated via three main pathways: classical, lectin, and alternative. Activation by the classical pathway is triggered through immunoglobulin (Ig) M/IgG, C-reactive protein (CRP), or apoptotic cell interaction with circulating C1q. IgM circulates as a hexamer, and upon binding to an antigen or pathogen surface, stabilizes with C1q binding to a single IgM molecule. Complementary activation via IgG is dependent on the density of IgG bound to antigen. Once bound to either IgM or IgG, C1q undergoes a confirmation change that results in the activation of C1r and the cleavage of C1s. C1s in turn cleaves C4 and C2 and activates C4b and C2a to generate the classical pathway C3 convertase. C3 convertase (C4bC2a) cleaves C3 into C3a and C3b. The second pathway, the lectin pathway, is triggered through recognition of microbial carbohydrates by mannose-binding lectin (MBL), collectins, or ficolins. The lectin pathway utilizes the same activated complement proteins as the classical pathway to generate classical pathway C3 convertase (C4bC2a). Both classical and lectin can also be activated by ischemia and reperfusion. The third pathway, an alternative pathway, is the dominant complement pathway due to its constitutive expression at low levels. The alternative pathway is activated by the spontaneous hydrolysis of C3 to generate C3(H_2_O). C3(H_2_O) will bind to factor B, which is then cleaved by factor D to generate an alternative pathway C3 convertase (C3bBb). Both classical and alternative C3 convertases catalyze the proteolysis of C3 into C3a (anaphylatoxin) and C3b. C3 convertase is stabilized by properdin, a molecule that is released by macrophages and T cells. The activation of C3b leads to the generation of C5 convertase, whose components differ based on which pathway is activated. Several other pathway-specific differences in C3 convertases also exist (reviewed in [80]). The classical and lectin pathway C5 convertase complex consists of C4bC2aC3b, while an alternative pathway C5 convertase consists of C3b2Bb. Both C5 convertases cleave C5 to C5a and C5b.

The generation of C3a and C5a, which is commonly referred to as anaphylatoxins, causes inflammation through increasing vascular permeability and attracting immune cells. Both C3a and C5a can cause reactive oxygen bursts in immune cells and pro-inflammation cytokine release [81,82,83]. C3a and C5a bind to their respective receptors, C3aR and C5aR, on the surface of myeloid cells [84,85,86,87,88] and non-immune cells [89,90,91]. However, in the plasma, both C3a and C5a are quickly converted to C3a desArg and C5a desArg. C3a desArg does not bind to its receptor, while C5a desArg does not exhibit the same inflammatory properties as C5a. C3a has both pro-inflammatory and anti-inflammatory properties. Upon binding to their respective receptors, C3a-C3aR results in the activation of protein kinase B and mitogen-activated protein kinase pathways [92] and IL-6 production [93], TNFα [94], and IL-1β [95]. C5a binding to C5aR results in the activation of phosphoinositide 3-kinase-γ and mitogen-activate protein kinase kinase pathways. Both C5a and C5a desArg can bind to two receptors, C5aR and C5L2, although with different affinities. C5a bound to C5aR on myeloid cells results in inflammatory cytokine production, while conversely, C5a binding to C5L2 appears to negatively regulate neutrophil function [96].

C3b also acts as an opsonin by covalently binding to hydroxyl groups on cell surfaces. C3b has a higher affinity to hydroxyl groups on pathogen cell surfaces; these are mainly carbohydrates, along with C4b and IgG. C3b binds to several complement components: C5, complement receptor 1 (CR1), and complement factors B, H, and I. Factor I along with cofactors C4BP, CR1, decay-accelerating factor, membrane cofactor protein, and complement factors B and H lead to C3b cleavage into iC3b and subsequent inactivation [97] along with C4b. Complement factor H has specific activity for C3b, while C4BP inactivates C4b. Properdin from degranulated neutrophils has been shown to recruit C3b and contribute to alternative pathway activation [98]. Due to the constitutive low-level expression of alternative pathway C3, complement inhibitors including complement factors I, H, and CR1 are found in circulation. CR1 is a cofactor of complement factor I that cleaves iC3b to generate C3c and C3dg. Both complement factor H-related protein 4A and P-selectin have been shown to aid in C3b binding to the cell surface [99,100]. In the event that C3b attaches to the surface of host cells, membrane-bound complement regulators prevent the destruction of the cell. In the instance of apoptotic cells, the expression of these complement regulators is decreased to promote immune-mediated clearance. C3b is required for the formation of the membrane attach complex (MAC). MAC forms a membrane pore that results in a calcium influx, resulting in cell death. MAC deposition plays a role in mitogenic signaling and cytokine release. Autologous cells are protected from MAC-mediated attack through an array of complement regulators, including CD59, a glycosylphosphatidylinositol (GPI)-linked membrane protein that restricts MAC formation. CR1 expressed on leukocytes contains extracellular complement control protein (CCP) domains that contain the binding sites for C3b and C4b [101]. CR1 is responsible for the iC3b cleavage into C3c and C3dg.

C3b binding to C3 convertases (both classical and alternative) increases the affinity for C5 convertases to bind to C5 [102,103]. C5 convertases will cleave C5 into two active products, C5a and C5b. C5a is considered a potent chemotactic molecule, and once it is bound to its receptor (C5aR) on neutrophils, macrophages and monocytes will increase the adhesiveness and evoke aggregations of neutrophils by upgrading adhesion molecule expression, increasing the oxidative phosphorylation (OXPHOX) rate, and macrophages and neutrophils secreting lysosomal enzyme. C5b undergoes a conformational change and induces opsonization through binding with C6 and C7. C5b, C6, and C7 associate with the cell membrane of a targeted cell. Afterwards, C8 and C9 bind to form the MAC. CR1 can also dissociate C5 convertase.

### 4.2. Role of Complement Activation during I/R Injury

I/R injury is the primary source of liver damage during procedure for transplantation. Liver damage manifests as hepatocyte loss through apoptosis and necrosis, leading to impaired graft function, which is confirmed by elevated liver enzymes aspartate transaminase (AST) and alanine transaminase (ALT). Several graft factors, including cold/warm ischemic time, donor age, and the degree of steatosis can affect the magnitude of I/R injury. During ischemia/reperfusion injury, all three pathways of the complement system can be activated, resulting in activated C3 and C5, triggering a sterile inflammatory response and MAC deposition. MAC was detected in transplant reperfusion-phase biopsies and correlated with elevations in AST as well as leukocyte and platelet infiltration [104]. In a rat model of I/R injury, transplanted livers deficient in the MAC component C6 contained less pro-inflammatory immune cells and cytokines, as well as less expression of pro-apoptotic protein caspase-3, implicating a MAC downstream of the complement activation in I/R injury [105].

Classical pathway components C4d-CRP and C3d-CRP were found to be activated in patients undergoing partial hepatectomy [106]. The complement components CRP, MAC, and C3 were co-localized in a rat I/R model [107]. IgM and CRP both activate the classical pathway through binding of C1q, shown to activate the complement pathway in a rat I/R model [108]. Complement also activated Kupffer cells [40], leading to inflammatory cytokine and ROS production [109]. Renal I/R injury models have suggested that IgM is involved in the activation of the lectin pathway [110,111]. Additional evidence has shown that IgM binds to apoptotic cells and aids in uptake by phagocytic immune cells rather than complement activation [112]. The precise role of IgM in I/R injury-induced complement activation remains under investigation.

CD59, a cell-surface receptor, inhibits C9 binding and prevents MAC formation. CD59 knockout mice, lacking expression of the endogenous MAC complex inhibitor, trended toward elevated AST/ALT compared with wild-type mice, which a double knockout of C3 with CD59 confirmed to be complement-dependent [113]. IL-6 and TNFα production were amplified with CD59 deficiency, indicating that MAC-mediated cell lysis may be an important step in early-phase endothelial dysfunction and late-phase reperfusion injury mediated by neutrophil accumulation and activation [113]. 

### 4.3. Strategies Targeting Complement Activation in I/R Injury

Several strategies targeting complement have been investigated in animal models, with the goal of reducing the magnitude of I/R injury. The C1 inhibitor (C1-INH) inhibits both the classical and lectin pathways through C1s and C1r and MASP-1 and 2, sparing the alternative pathway. When C1-INH was added to cold preservation solution, it decreased C3 deposition in animal models of ex vivo liver reperfusion [114]. The administration of C1-INH prior to ischemia in a rat I/R model decreased liver injury, AST/ALT elevations, as well as leukocyte and platelet aggregation [107,115]. Similar results were obtained in a mouse hepatic I/R injury model [116].

Soluble CR1 (sCR1) inhibits complement activation via the classical and alternative pathways, and was shown to be effective in a rat model of ischemic myocardium [117] and mesenteric atrial occlusion [118]. The administration of sCR1 immediately before reperfusion reduces leukocyte adherence to sinusoids, liver enzymes, and hepatocyte death in rat I/R models [119,120]. Rats that underwent I/R injury showed decreased C3 deposition in the liver after the administration of sCR1 [121]. The effect of sCR1 mirrored that of animals depleted of complement with cobra venom factor, and specifically reduced polymorphonuclear leukocyte accumulation [40]. Complement inhibition with sCR1 also decreases Kupffer activation, neutrophil accumulation, microvascular dysfunction, and injury after hepatic I/R injury [119,120,121]. 

C5aR antagonists that aimed at reducing recruitment and activation of immune cells have been investigated in I/R injury models. C5aR antagonists were effective in decreasing serum AST and tissue levels of TNFα and myeloperoxidase in kidney I/R injury animal models [122]. Similar results were obtained in rat hepatic I/R injury, in which a C5aR antagonist lowered liver enzymes, as well as decreased serum, tissue TNFα, and neutrophil infiltration [123].

Rodent-specific complement inhibitors have been developed to decrease systemic complement inhibition and bacterial susceptibility. Complement receptor 2 (CR2) is found on the surface of mature B cells and binds to activated C3, which include iC3b, C3dg, and C3d. CR2-deficient mice are protected from intestinal mouse I/R injuries [124]. CR2-Crry, a complement inhibitor fusion protein consisting of CR2 and a CR1-related gene (Crry), showed increased survival and marginally affected serum complement levels in an intestinal I/R injury model of sepsis [125].

During sterile inflammation, IgM antibodies bind to redox-induced neoepitopes, triggering complement activation. IgM binding to neoepitopes in Annexin IV, and phospholipids triggered complement activation and I/R injury in Rag1^-/-^ mice, which are protected from I/R injury [126]. Restoring complement activation via IgM amplified liver injury, as evident by elevated serum ALT levels. Blocking IgM binding to Annexin IV reduced serum ALT and improved histology scores following I/R. The modified Annexin IV antigen was observed in ischemic donor liver tissue, with elevated IgM and C3d deposition. This data suggests that neoepitope recognition may be the critical event linking the ischemia and reperfusion phases of I/R injury.

## 5. Role of Donor Liver Steatosis in Complement Activation

Insulin resistance, obesity, and other metabolic disorders are well-known risk factors for NAFLD development. The progression from benign simple steatosis to steatohepatitis is marked by shifts in glucose/lipid metabolism and antioxidant imbalance, which directly affect pro-survival and stress response pathways and can lead to hepatocyte turnover and inflammation. Hepatocyte turnover and the resulting inflammatory response signal the progression of NAFLD to non-alcoholic steatohepatitis (NASH). However, recent studies have demonstrated peripheral inflammation in NAFLD, including elevations in serum C3 [127,128,129]. Serum C3 elevation was recently proposed and validated as a biomarker predicting NAFLD independent of obesity and metabolic co-morbidity [130,131]. Several products of complement activation have been observed in the liver biopsies of NAFLD patients, specifically C4d, C1q, MBL, and MAC [128,132]. The degree of complement activation product in the liver correlated with NAFLD disease severity, with increased deposition possibly identifying the earliest stages of hepatocyte lipoapoptosis. In addition to the increased baseline expression of complement components, circulating levels of the activation product C3a were shown to directly correlate with the percentage of hepatic steatosis, particularly as steatosis progressed from absent to moderate [133]. An increased expression of complement components and/or the presence of complement activation products may hold the key to identifying donor livers at the risk of steatosis-driven functionality impairments following transplantation.

These clinical observations have been supported by numerous studies using animal models of steatosis. Hepatocytes are the primary biosynthesis site for complement components [134,135], with increased production rapidly induced during inflammation. The cytokine-signaling pathways tied to IL-6 and IL-1β have been directly linked to increased C3 expression [136,137]. Complement components C3 and C5 may also have direct roles in lipid catabolism. A diet-induced model of NAFLD with simple steatosis in C3 knockout mice did not observe a difference in the onset or degree of steatosis [73], although postprandial triglyceride clearance is delayed [138]. Conversely, C5 knockout mice had delayed steatosis and triglyceride accumulation following high-fat diet feeding, which was accompanied by early increases and late decreases in TNFα and IL-6 kinetics [139].

**Figure 1:**
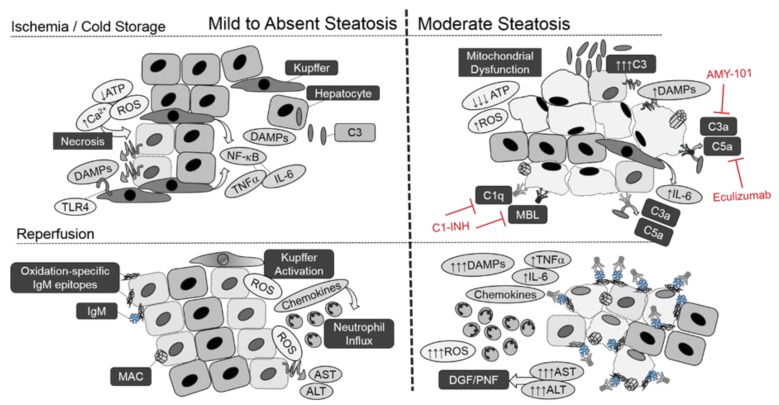
Overview of Aberrant Complement Activation in Hepatic Steatosis.

Limited studies using these models have confirmed a direct role of complement in exacerbated response to I/R or transplant injury. In a diet-induced model of simple steatosis without steatohepatitis, C3 deletion improved 24-h survival from 33% to 100% [73]. A translational effect was confirmed by recipient treatment with CR2-Crry, deploying the C3 inhibitor Crry directly to sites of C3 opsonization by means of a fusion protein containing the iC3b/C3dg-binding fragment for CR2. The results of this study suggest that the exacerbated injury observed in liver steatosis may be due to increased neoepitope exposure, resulting in increased complement-mediated hepatocyte turnover. A recent study showed that B1a cells lacking Siglec-G are enriched in the production of natural IgMs, with specificity directed against oxidation-specific epitopes, such as oxidized low-density lipoprotein and dying cells [140,141,142,143,144,145,146,147,148]. Oxidative-specific epitopes have already been implicated in NAFLD progression to NASH [149,150]. The IgM axis is also involved in the clearance of apoptotic cells through C1q [151]. Collectively, these studies suggest a prominent role for a primary, IgM-mediated complement mechanism driving reperfusion injury as well as its exacerbation by underlying steatosis. This is supported by the significant drop in post-reperfusion ALT in both C3 and C2-Crry treated steatotic mice. The results were confirmed in an orthotopic liver transplant model in mice, where CR2-Crry reduced reperfusion ALT levels in both lean and steatotic mice. Inhibiting complement with CR2-Crry also dampened downstream mediators IL-6, TNFα, and neutrophil infiltration, which are all known to play a role in late-phase reperfusion injury. Although the deposition of MAC was confirmed in NAFLD biopsies [128], there are limited studies on the role of MAC in I/R or transplant injury with underlying steatosis. The studies support a role of complement activation in the progression of steatosis to steatohepatitis, wherein the degree of complement involvement may provide a key biomarker for susceptibility to I/R injury. An overview of complement activation in steatosis is provided in Figure 1.

When macrosteatosis is minimal, the ischemic and cold storage phase triggers metabolic adaptation, resulting in decreased ATP production, Ca^2+^ influx, and increased redox species production through defective oxidative phosphorylation. If the hypoxic injury is sufficient to compromise the hepatocyte, DAMPs, such as HMGB1, are released and signal through TLR4-NF-κB to generate a sterile inflammatory response. During reperfusion, continued ROS accumulation and the activation products of Kupffer cells and neutrophils cause a secondary phase of hepatocyte death, resulting in elevated liver enzymes (AST and ALT). Complement activation may play a greater role when macrosteatosis is moderate to severe. Lipid-laden hepatocytes display impaired ATP production due to mitochondrial dysfunction and increased C3 synthesis at baseline. Early complement activation is also apparent prior to injury, which may be linked to low levels of lipotoxicity in hepatocytes. The ischemia and storage phase results in increased ROS levels, DAMP release, and inflammatory cytokine production in conjunction with increased hepatocyte turnover. Complement component deposition prior to and during ischemia results in aberrant complement activation, which is further increased by the binding of IgM to apoptotic cells and oxidation-specific epitopes. Hepatocyte cytotoxicity approaches catastrophic levels due to the combined influence of redox species, MAC-dependent cell lysis, and the activation products of Kupffer cells and neutrophils, resulting in delayed, or in extreme cases, primary graft dysfunction.

## 6. Role of Complement in Liver Donation after Brain Verses Cardiac Death

Brain death increases pro-inflammatory molecules, resulting in an autonomic storm causing hormonal differences, hemodynamic instability, and a cascade of inflammatory processes that raise both blood C3 levels and local complement, which ultimately affect transplant outcomes. Studies in kidney transplantation have revealed that both brain and cardiac death significantly increase C3 expression and complement activation compared with living donors [152,153,154,155]. Administering steroids prior to transplantation has proved to be an effective anti-inflammatory treatment, although it does not appear to affect increased C3 expression in animal models of deceased donors [156,157]. The role of complement activation following brain death, and targeting complement to reduce transplant injury, have been well reviewed [158]. Studies that compare the role of complement in donation after brain death (DBD) verses donation after cardiac death (DCD) are limited in solid organ transplantation are extremely limited, particularly in liver transplantation.

## 7. Steatosis in Living Donors

Steatosis levels for living liver donations are difficult to assess prior to surgery. While the best method to assess steatosis is through biopsy, non-invasive methods such as computed tomography and magnetic resonance imaging scans are routinely used for living donors. The increasing prevalence of NAFLD in the population heightens the necessity of determining steatosis levels in living donors. A study by Ryan et al. showed that steatosis was present in 33% of living donor livers [159]. The presence of MiS only in living donor livers did not affect post-transplant survival [160]. One advantage to live donations is the short cold ischemia time, and thus damage accumulated during I/R will be reduced compared to OLT. The percentage of steatosis that is deemed acceptable to use remains up to the discretion of transplant centers. 

## 8. Targeting Complement Activation in Liver Transplantation

Several advancements and promising investigational approaches have emerged in liver transplantation. New formulations and modifications to storage solutions have shown promise in preserving organ function and preventing static storage I/R injury. Unfortunately, these investigations have primarily focused functional assessments on post-transplant outcomes and laboratory values. Whether recent advancements in storage solutions have modulated complement activation remains unclear. A 1994 study shows that the expression of complement regulatory proteins–membrane cofactor protein, decay accelerating factor, protectin, and CR1 were unaffected by storage in University of Wisconsin solution [161]. Two machine perfusion safety and feasibility trials are underway, supported by several years of preclinical work. As with preservation solutions, endpoints have focused on endpoint liver enzymes in lieu of post-perfusion organ analysis or early markers of reperfusion injury.

### Complement-Directed Therapeutics in Liver Transplant I/R Injury

The use of complement inhibitors to target I/R injury in liver transplantation is in its infancy. Several strategies to treat antibody-mediated rejection through complement inhibition are under investigation (Table 1), which may have important implications in reducing I/R injury and DGF. Eculizumab, a humanized monoclonal antibody inhibiting C5 cleavage to C5a and C5b, is under active investigation for antibody-mediated rejection in kidney transplantation. The PROTECT study, a phase 2/3 study to prevent delayed graft function in kidney transplantation, did not reach its primary endpoint of improving DGF rates. However, a recent single-center randomized trial of eculizumab in pediatric kidney transplant recipients showed improved early graft function and morphology, but with an unacceptably high number of graft losses related to unvaccinated influenza infections [162]. The trial by Kaabak et al. [162] utilized an early dosing regimen based on pharmacodynamic analysis, which may be a better approach to complement neutralization.

C1-INH, a plasma-purified classical and lectin pathway inhibitor preventing C2 and C4 formation, was shown to be safe and effective in kidney transplant patients, successfully reducing antibody-mediated rejection [163]. A more recent trial investigating C1-INH in preventing DGF in kidney transplant patients showed that intraoperative C1-INH reduced post-transplant dialysis requirements and improved renal function at one-year endpoints, with patients at a preoperative risk of DGF showing the greatest benefit [164].

Compstatin, a C3-binding synthetic peptide, was shown by Nilsson et al. to bind C3, effectively preventing cleavage to C3a and C3b [165]. AMY-101 is a next-generation Compstatin analogue with increased affinity, inhibitory potency, and half-life [166]. AMY-101 is under first-in-human trial evaluation, and holds promise as a small molecule approach to neutralizing complement to prevent DGF in transplantation.

## 9. Conclusions

It is clear that the rising incidence of NAFLD will impact the donor pool for liver transplantation. Several retrospective studies have helped support the <30% and >60% MaS thresholds for donor steatosis, although several reports have demonstrated safe utilization of these organs with comparable outcomes. Although cold ischemia time appears to be critical in utilizing livers with elevated MaS, there is no current strategy that distinguishes low verses high risk within MaS tiers. The sole use of MaS tiers for procurement will continue to limit utilization. The role of complement activation as the primary driver of aberrant I/R injury in steatotic livers is becoming clearer. Complement component levels in the circulation and donor biopsy may provide a valuable resource in confirming elevated risk in MaS. Donor livers with MaS with mild complement expression or activation may be excellent candidates for complemented-directed therapy to reduce MaS risk. As machine perfusion strategies advance, prophylactic complement administration may be an effective strategy to salvage MaS donor organs at risk of complement-mediated DGF. A thorough evaluation of temporal complement in liver regeneration following transplantation is required to tailor complement inhibition protocols to yield optimal patient outcomes. 

## Figures and Tables

**Table 1:** Liver transplant therapeutics targeting complement.

Molecule		Target	Effect	References (Clinical Trials)
Eculizumab	Humanized monoclonal antibody	C5	Prevents C5 cleavage	[155] (NCT1756508)
C1-INH	Plasma purified protein	C1s	Prevents C2 and C4 formation	[156,157] (01134510)
AMY-101	Synthetic peptide	C3	Prevents cleavage of C3	(NCT03316521)

## Abbreviations

I/R	Ischemia/Reperfusion
ECD	Extended Criteria Donor
DGF	Delayed Graft Function
PNF	Primary Non-Function
MiS	Microsteatosis
MaS	Macrosteatosis
NAFLD	Non-Alcoholic Fatty Liver Disease
ROS	Reactive Oxygen Species
DAMP	Damage-Associated Molecular Pattern
HMGB1	High-Mobility Group Protein 1
TLR4	Toll-Like Receptor 4
TNFα	Tumor Necrosis Factor α
iNOS	Inducible Nitric Oxide Synthase
NF-κB	Nuclear Factor Kappa-Light-Chain-Enhancer of Activated B Cells
HO-1	Heme-Oxygenase 1
Nrf2	Nuclear Factor Erythroid 2
PPAR	Proliferator-Activated Receptor
Ig	Immunoglobulin
AST	Aspartate Aminotransferase
ALT	Alanine Aminotransferase
OLT	Orthotopic Liver Transplantation
MAC	Membrane Attack Complex
MBL	Mannose-Binding Lectin
DBD	Donation After Brain Death
DCD	Donation After Cardiac Death
NASH	Non-alcoholic Steatohepatitis
